# Editorial: Advances in primary immunodeficiencies (inborn errors of immunity) in Central-Eastern Europe, volume II

**DOI:** 10.3389/fimmu.2023.1221137

**Published:** 2023-06-13

**Authors:** Malgorzata Pac, Jean-Laurent Casanova, Irina Tuzankina, László Maródi

**Affiliations:** ^1^ Department of Immunology, The Children’s Memorial Health Institute, Warsaw, Poland; ^2^ St. Giles Laboratory of Human Genetics of Infectious Diseases, The Rockefeller University, New York, NY, United States; ^3^ Howard Hughes Medical Institute, New York, NY, United States; ^4^ Laboratory of Human Genetics of Infectious Diseases, Necker Branch, Necker Hospital for Sick Children and Institut National de la Sante et de la Recherche Medicale (INSERM), Paris, France; ^5^ University Paris Cité, Imagine Institute, Paris, France; ^6^ Institute of Immunology and Physiology of Ural Branch of the Russian Academy of Sciences, Yekaterinburg, Russia; ^7^ Department of Dermatology, Primary Immunodeficiency Clinical Unit and Laboratory, Semmelweis University, Budapest, Hungary

**Keywords:** SCID (severe combined immunodeficiency), inborn error immunity, primary immunodeficiencies, antibody deficiency, newborn screen (NBS), JProject, education, awareness

Knowledge of Primary Immunodeficiencies (PIDs) or Inborn Errors of Immunity (IEI) has expanded exponentially during the last 20 years. New and improved diagnostic tools, including new generation sequencing (NGS), and gene product activity or functional tests have led to profound advances in clinical immunology. These advances have made it easier to understand the mechanisms and pathogenesis of different types of IEI, and subsequently to introduce targeted therapies. They help define and recognize the increasing number of IEIs. It is worth noting the tremendous role of the International Union of Immunological Societies (IUIS) and its Expert Committee in updating the categorization of IEI approximately every 2 years. According to the last report in 2022, 485 different genetic defects causing IEI are known (55 novel compared to the 2019 report), previously categorized into 10 groups and published as phenotypical classification as well. Taking into account that number of all IEIs remains underestimated, the efforts of enthusiastic clinicians and scientists to improve diagnostics, recognition, and treatment are of great value (1–3). Much of this progress in the field of clinical and experimental immunology has been made in recent decades, mainly in western Europe, the USA, Japan, and Australia (1–5), with significant contributions also coming from Latin America, Africa, the Middle East, and both South and East Asia. Eastern and Central European (ECE) countries were isolated for many years, until the fall of the iron curtain in 1989, with limited access to the newest scientific achievements, diagnostic tools, and therapeutic methods. Only personal connections, and direct collaboration with clinical and research centers in Western Europe and the US, made some progress possible in that region. Within the last 30 years, there has been a great deal of effort to overcome the gap between ECE and Western Europe in terms of IEI diagnostics, including molecular tests, treatment, and education. One of the most important initiatives was the J Project, initiated in 2004 by clinicians and scientists in Eastern and Central Europe. The goal of the JProject (JP) was to increase awareness, facilitate diagnosis including genetic tests, and improve therapy according to the latest knowledge in the area of the ECE region. In subsequent years, collaboration expanded to include “daughter J Projects” in countries such as Turkey, Iran, Egypt, Russia, and others (5–10).

Between the end of 2019 and 2020, we successfully published 11 manuscripts as an e-book dedicated to “*Advances in Primary Immunodeficiencies (Inborn Errors of Immunity) in Central-Eastern Europe*”, covering the results of clinical and scientific work in the separate countries in the region as well the effects of the JP network (11). One year later we decided to edit the second volume of a special Research Topic on “*Advances in Primary Immunodeficiencies (Inborn Errors of Immunity) in Central-Eastern Europe*” to expose the successful efforts of single immunological centers or countries as well as the effects of scientific collaboration within the ECE region and western Europe and/or the US in the field of IEI. An invitation was sent by the Editors of Frontiers in Immunology to our colleagues from ECE and JProject collaborative immunological centers to submit original research articles, commentaries, opinion, and review articles resulting from the mentioned collaboration and documenting experiences covering areas such as the molecular defects of PIDs, achievements in diagnostics, the clinical characteristics of different PIDs, region-specific PIDs, the current treatment of different PIDs with immunoglobulin replacement therapy (IgRT), hematopoietic stem cell transplantation (HSCT), and biological treatment of autoinflammatory diseases.

After the review and editorial process, this Research Topic includes 19 articles reflecting new diagnostic tools, their influence on the recognition of IEI, country-related registries, and analysis of the clinical course of known and novel IEI and mutations as well as which treatments were selected for publications.

In March 2020, the World Health Organization (WHO) declared a COVID-19 pandemic, which has affected people all over the world regardless of age, sex, or comorbidities, with worse prognosis for older people with type I interferon autoantibodies or concomitant diseases. Patients with IEI also appeared to be at higher risk of developing COVID-19 at the start of the pandemic. However, further observations from different centers have shown that only certain types of IEI are associated with poor prognosis (12–19). It was not surprising to receive reports on the course of COVID-19 and the role of vaccination against SARS-CoV-2 in IEI patients in ECE. A Polish study on a group of 150 patients (adults, adolescents, and children) published by Koltan et al. shows that in severe humoral defects (CVID and XLA), the risk for a severe course of COVID-19 increases significantly with age and comorbidities. It also indicated the need for vaccination against SARS-CoV-2 within that group. Despite earlier reports on humoral and cellular response to immunizations against COVID-19 in inborn defects of immunity (20, 21). Another Polish group assessed SARS-CoV-2 vaccination coverage and hesitancy in adults with primary immunodeficiencies and autoinflammatory and rheumatic diseases on biologic therapy (Więsik-Szewczyk et al.). They showed a higher percentage of vaccinated individuals in each group compared to the general population in Poland.

Some papers stress the relevant role of awareness of IEI across countries as well as collaboration between immunological centers of the J Project. Naumova et al. demonstrated a higher number of newly diagnosed IEI in Bulgaria as a result of awareness, as well as the establishment of Expert Centers for Rare Diseases and the creation of national registries. Eldeniz et al. suggests adding the history of parental consanguinity and tuberculosis in the family to the list of warning signs of primary immunodeficiencies originally developed by the Jeffrey Modell Foundation. Taking into account the high percentage of consanguineous marriages in some nations or communities as well as the risk of tuberculosis is noteworthy. An accurate and significant conclusion on the tremendous role of cooperation between medical professionals in JP countries on the development of diagnostics, treatment, education, and awareness in the field of IEI is presented by Abolhassani et al.

In a mini review, Nikolouli et al. provide an analysis of the currently available *in vitro* models used to study IEI and its role in new therapeutic approaches. Following the world trend to encourage the timely diagnosis of severe combined immunodeficiencies as well as other severe T or B lymphopenia, pilot study results from Ukraine were published. TREC and KREC analyses were undertaken for almost 10 500 newborn children for severe combined immunodeficiency (SCID) and other severe IEI, with one case of CID detected. The DNA samples from known IEI (SCID, CID, XLA) were used as controls. The study proved that newborn screening for SCID and other severe IEI and proper treatment procedures can be introduced, such as HSCT and IgRT before the first symptoms and complications occurr. Select papers describe the new genetic causes of different inborn errors of immunity, such as STIM1 GOF mutation, and GATA2 defect. Other reports concern rare and not typical manifestations of known gene defects, including LIG1 deficiency and Omenn-like syndrome, SRP54 deficiency, and cyclic neutropenia or *Pneumocystis jirovecii* pneumonia in Aicardi-Goutieres syndrome. Different clinical aspects, such as pulmonary lesions or sleep quality or fatigue assessment in adult patients with humoral defects and other primary immunodeficiencies have been discussed by Polish authors. The essential issues of advancement in early diagnostics and treatment of ADA-SCID patients are presented in a multicenter report from Poland, which showed the limitations of enzyme replacement therapy due to its high costs and lack of approval in the EU, and restricted access to gene therapy. This indicates that the implementation of a newborn children screening program for SCID in Poland could improve early recognition and treatment of all SCID, including ADA-SCID. There was also scope to discuss experimental aspects of human hepatocyte transplantation for liver disease.

It should be emphasized that the papers published in this Research Topic document a substantial improvement in IEI awareness and research in Central and Eastern Europe. This increased awareness of IEI has led to better and quicker recognition, including new gene mutations in different IEI, introducing new diagnostic tools and treatment approaches, as well as a better understanding of IEI-related complications or mechanisms, as shown in the articles included in this Research Topic.

The Editors hope that the second edition of this Research Topic on “*Advances in Primary Immunodeficiencies (Inborn Errors of Immunity) in Central-Eastern Europe*” will allow readers to learn more about the remarkable developments in the ECE region in terms of IEI-related specific diseases, their molecular background, and novel mutations in different phenotypes. It should also stimulate further research and cooperation within the J Project in ECE countries and elsewhere in this rapidly developing field of molecular medicine. Finally, we are grateful to the Editors of Frontiers in Immunology for their invitation to bring this collection together.

## Author contributions

MP, J-LC, and LM contributed to the work equally. MP, J-LC, LM, and IT read and approved it for publication.
